# Serotype Distribution and Antimicrobial Resistance Profile of *Haemophilus influenzae* Isolated from School Children with Acute Otitis Media

**DOI:** 10.1155/2022/5391291

**Published:** 2022-05-23

**Authors:** Dodi Safari, Daniel Joko Wahyono, Wisnu Tafroji, Anton Budhi Darmawan, Yayah Winarti, Wahyu Dwi Kusdaryanto, Wisiva Tofriska Paramaiswari, Hendro Pramono, Meyta Pratiwi, Muhamad Riza Chamadi

**Affiliations:** ^1^Eijkman Institute for Molecular Biology/Pusat Riset Biologi Molekuler Eijkman, Cibinong, West Java, Indonesia; ^2^Jenderal Soedirman University, Purwokerto, Indonesia; ^3^Department of Otorhinolaryngology, Head and Neck Surgery, Jenderal Soedirman University, Purwokerto, Indonesia

## Abstract

*Haemophilus influenzae* is a Gram-negative opportunistic bacterial pathogen of the human respiratory tract. This study describes the prevalence, serotype distribution, and susceptibility profiles of *H*. *influenzae* strains isolated from the nasopharynx of school children with acute otitis media (AOM) in Banyumas Regency, Central Java, Indonesia. *H*. *influenzae* was isolated from nasopharyngeal swab specimens using chocolate agar plates supplemented with IsoVitaleX and bacitracin. Serotyping was performed using quantitative polymerase chain reaction. Antimicrobial susceptibility profiles were determined using a microdilution broth assay. *H*. *influenzae* was present in 69.7% of samples (85/122). Nontypeable *H*. *influenzae* (NHTi) was the most common serotype (95.3%), followed by *H*. *influenzae* type b (3.5%) and *H*. *influenzae* type *f* (1.2%). All the *H*. *influenzae* isolates were susceptible to levofloxacin, ceftriaxone, imipenem, meropenem, cefuroxime, and cefixime. Most isolates were susceptible to sparfloxacin (99%), cefepime (99%), amoxicillin/clavulanic acid 2 : 1 (99%), ampicillin/sulbactam 2 : 1 (96%), chloramphenicol (94%), tetracycline (93%), ampicillin (87%), and clarithromycin (82%). Nineteen percent of the isolates were resistant to cotrimoxazole, and 11% of the isolates were resistant to ampicillin. This study showed that *H*. *influenzae* carriage among samples was dominated by NTHi and less susceptible to cotrimoxazole.

## 1. Introduction

*Haemophilus influenzae* is a Gram-negative opportunistic bacterial pathogen of the human respiratory tract. This bacterium is grouped into capsulated and noncapsulated bacteria (nontypeable *Haemophilus influenzae*; NTHi) [1, 2]. Noncapsulated bacteria have been reported as the most common pathogenic bacteria causing invasive disease since the implementation of the Hib vaccine. *H*. *influenzae* contributes to approximately 21,000 otitis media-associated deaths annually [3]. The NTHi strain mainly colonizes the mucosal surfaces of the upper respiratory tract and is highly associated with acute otitis media (AOM), sinusitis, bronchitis, exacerbations, and chronic persistent infections in older patients with chronic obstructive pulmonary disease [4]. NTHi is one of the three dominant bacterial otopathogens causing otitis media, which has been reported globally along with other pathogens such as *Streptococcus pneumoniae* and *Moraxella catarrhalis* [5]. The presence of *H*. *influenzae* in the nasopharynx was associated with older age and recurrent AOM. The proportion of NTHi-causing otitis media has trended upward in the postpneumococcal conjugate vaccine (PCV) era, and the majority of NTHi isolates were nonsusceptible to ampicillin in Taiwan [6]. Recently, NTHi strains isolated from the nasopharynx of HIV-infected patients were less susceptible to ampicillin (62%) and trimethoprim/sulfamethoxazole (cotrimoxazole) (41%) in Indonesia [7]. More than half of the *H*. *influenzae* strains isolated from pediatric patients with AOM in Japan are genotypic *β*-lactamase-nonproducing ampicillin-resistant strains [8]. Studies on *H*. *influenzae* among school-aged children with AOM in Indonesia are limited. In this study, we investigated the serotype distribution and susceptibility profiles of *H*. *influenzae* strains isolated from the nasopharynx of school children with AOM in Banyumas Regency, Central Java, Indonesia.

## 2. Methods

### 2.1. Specimen Collection

*H*. *influenzae* was isolated from school children with AOM aged 6 to 12 years in Banyumas Regency, Central Java, Indonesia. Specimen collection was performed for 13 months, from September 2018 to October 2019. The study was reviewed and approved by the ethics committee of the Faculty of Medicine, Jenderal Soedirman University, Purwokerto, Indonesia, No. 4015/KEPK/FK/2018.3574. Among school children screened for AOM infection, 166 children were diagnosed with positive cases of AOM [9, 10]. However, only 122 nasopharyngeal (NP) swab specimens were available for the present study. The NP swab specimens were collected with sterile FLOQSwabs (Copan) and inoculated into 1 mL STGG (skim milk (BD), tryptone (BD), dextrose (BD), and glycerol (Sigma)) as a transport medium. The specimens were then stored at −70°C before further testing.

### 2.2. *Haemophilus influenzae* Isolation and Identification

Briefly, the isolation was performed as follows: a 100 µL of inoculated STGG media with nasopharyngeal swab specimens were streaked on chocolate agar plate supplemented (sCAP) with IsoVitaleX (BD) with addition of bacitracin (20 U ml^−1^) (Sigma), followed by incubation at 37°C with 5% CO_2_ for 20 h. Extended incubation for 48 h was performed for no-growth plates. All suspected *H*. *influenzae* isolates were identified by Gram staining (BD) for Gram-negative coccobacillus, oxidase test, and XV factor-dependent test (Oxoid) [7].

All suspected *H*. *influenzae* isolates were further confirmed using quantitative polymerase chain reaction (qPCR) targeting hpd encoding *H*. *influenzae* protein *D* (Table 1), as described previously [11]. All isolates were subcultured onto a sCAP and incubated at 37°C in a 5% CO_2_ atmosphere for 20 h. The DNA was extracted using a boiling method as follows: a fine touch of single colony was transferred into 200 *µ*L Tris-EDTA (Sigma), then homogenized, and heated at 100°C for 5 min. The suspension was then immediately incubated at −20°C for 5 min, followed by centrifugation at 13,000 × *g* for 10 min. The qPCR reaction mixture consisted of TaqMan Universal Master Mix (Cat. No. 4304437), ROX 1 : 10 (25 *μ*M), paired hpd primers, and probe. The DNA template used for each reaction was 2.5 *µ*L. The *H*. *influenzae* ATCC 49247 strain was used as the positive control. The qPCR conditions were set as follows: 2 min at 50°C, followed by 95°C for 10 min as predenaturation, and 40 cycles at 95°C for 15 s and 60°C for 1 min. The hpd was considered positive if the Ct was ≤35; Ct ranging from 36 to 40 was repeated with dilution, and negative if Ct was >40 or defined as undetermined by the instrument.

### 2.3. Serotype Determination

Serotyping was performed using qPCR, as described previously [11].There were six single reactions for the detection of serotypes a (acsB), b (bcsB), c (ccsD), d (dcsE), e (ecsH), and f (bexD) using six pairs of primers and different probes according to the target gene to be amplified [11]. The qPCR conditions were similar to those for hpd detection.

### 2.4. Antimicrobial Susceptibility Testing

Antimicrobial susceptibility testing was performed using microdilution broth following the Clinical Laboratory Standard Institute (CLSI) guidelines using 96-well round-bottom MIC plates (Thermo Fisher Scientific, Cat. No. HPB1), containing 20 antimicrobials. ATCC 49247 *H*. *influenzae* was used as the quality control strain. The resistance level of *H*. *influenzae* was interpreted according to the CLSI 29^th^ edition breakpoints [12]. Further analysis of ampicillin-resistant isolates was performed with *β*-lactam resistance classification based on the presence of *β*-lactamase and on the values of MIC of ampicillin (AMP) and amoxicillin/clavulanate (AMC) : BLNAS, *β*-lactamase-nonproducing ampicillin susceptible (AMP MIC ≤1 *µ*g/mL), BLNAI, *β*-lactamase-nonproducing ampicillin intermediate resistant (AMP MIC = 2 *µ*g/mL), BLNAR, *β*-lactamase-nonproducing ampicillin-resistant (AMP MIC ≥4 µg/mL with nonproducing *β*-lactamase), BLPAR, *β*-lactamase-producing ampicillin-resistant (AMP MIC ≥4 *µ*g/mL and AMC ≤4/2 *µ*g/mL with *β*-lactamase), and BLPACR, *β*-lactamase-producing amoxicillin/clavulanate resistant (AMC ≥8 *µ*g/mL with *β*-lactamase) [13].

## 3. Results

We identified 69.7% (85/122) of school children with AOM positive for *H*. *influenzae*, with the majority of strains being NTHi (81/85; 95.3%), followed by capsulated *H*. *influenzae*, 3.5% (3/85) for type b, and 1.2% (1/85) for type *f* (Table 1). The antimicrobial resistance profiles of the 85 *H*. *influenzae* isolates revealed that all isolates were susceptible to several antibiotics (Table 2). The majority of the isolates were susceptible to sparfloxacin (99%), cefepime (99%), amoxicillin/clavulanic acid 2 : 1 (AMC) (99%), ampicillin/sulbactam 2 : 1 (96%), chloramphenicol (94%), tetracycline (93%), ampicillin (87%), and clarithromycin (82%) (Table 2). However, 19% (16/85) of the isolates were resistant to cotrimoxazole with MIC range, MIC50, and MIC90 as follows: ≤0.06/1.19 to >2/38 *µ*g/mL, 0.25/4.75 *µ*g/mL, and >2/38 *µ*g/mL, respectively. Moreover, 11% (9/85) of the isolates were resistant to ampicillin with MIC range, MIC50, and MIC90 as follows: ≤0.12 to >4 *µ*g/mL, 0.25 *µ*g/mL, and 4 *µ*g/mL, respectively. In addition, 18% (15/85) of the isolates were nonsusceptible to clarithromycin with MIC range, MIC50, and MIC90 as follows: ≤0.12 to 16 *µ*g/mL, 8 *µ*g/mL, and 16 *µ*g/mL, respectively. Meanwhile, less than 10% of the isolates were resistant to tetracycline, chloramphenicol, ampicillin/sulbactam, cefaclor, cefepime, sparfloxacin, and amoxicillin/clavulanic acid (Table 2). In this study, six isolates were defined as multidrug resistant (MDR) and were resistant to at least three antimicrobial classes.

Furthermore, the antibiogram of ampicillin nonsusceptible isolates was analyzed to determine *β*-lactamase-related resistance in the ampicillin nonsusceptible groups. This analysis resulted in 85% (72/85) of the isolates being defined as BLNAS. Interestingly, one isolate was defined as *β*-lactamase-nonproducing amoxicillin/clavulanate (2 : 1)-resistant (BLNACR), which showed resistance to amoxicillin/clavulanic acid (MIC value > 16/8 *µ*g/mL) but was susceptible to ampicillin. Also, 4% (3/85) of the isolates were defined as BLNAI, indicated by intermediate to ampicillin (MIC = 2 *µ*g/mL). Further, 7% (6/85) of isolates were resistant to ampicillin (MIC ≥4 *μ*g/mL). Moreover, three isolates that were *β*-lactamase-nonproducing ampicillin/sulbactam-resistant (BLNASR) were identified, and two isolates were resistant to cefaclor (Table 3).

## 4. Discussion

In this study, it was observed that 69.7% of the school children with AOM in Banyumas Regency, Central Java, Indonesia, tested positive for *H*. *influenzae* in their nasopharynx. NTHi was the major type (95.3%) observed in this study. The carriage prevalence of *H*. *influenzae* in this study was higher than that reported in previous studies (with an interval of 9–32%) in Indonesia [7, 14, 15]. Many previous studies reported correlation between bacteria colonizing the nasopharynx and otitis media cases [16–18]. Some bacteria colonizing the nasopharynx, including *H*. *influenzae*, *S*. *pneumoniae*, *and Moraxella catarrhalis* [16–18], and others such as *S*. *pyogenes* and *Staphylococcus aureus* were reported to be in concordance with those isolated from the middle ear fluid of patients with otitis media [18]. *S*. *pneumoniae* and *H*. *influenzae* were the most prevalent pathogenic bacteria that showed a positive association with otitis media infection. NTHi was reported as a common type of otitis media in many regions, including South America, North America, Germany, and Asia [18–20]. This is also concordant with the findings in this study, which defined 95.3% of *H*. *influenzae* isolates as NTHi. The implementation of the Hib vaccine in the national vaccine program might explain the high prevalence of NTHi among children in Indonesia. Detection of *H*. *influenzae*, which is dominated by NTHi, in the nasopharynx, showed 90.91% sensitivity for otitis media cases compared to middle ear fluid as the standard for determining otitis media etiological bacteria [17]. Furthermore, the development of otitis media due to the presence of pathogenic bacteria in the nasopharynx is also triggered by alterations in the nasopharynx environment caused by viral infection. The alteration of the nasopharyngeal environment, including ATP and glucose release from cell or tissue damage, norepinephrine release due to sympathomimetic response, and an increase in temperature, will induce dissemination and dispersal of pathogenic bacteria by inducing bacteria to produce bacteriocin and many virulence factors that upregulate the dispersal of bacteria [21].

Among 85 isolates of *H*. *influenzae*, it was discovered that 19% of isolates were resistant to cotrimoxazole followed by ampicillin (11%) while isolates resistant to tetracycline, chloramphenicol, ampicillin/sulbactam, cefaclor, cefepime, sparfloxacin, and AMC were less than 10%. This finding was in concordance with a study in Thailand which reported that strains of *H*. *influenzae* isolated from patients with otitis media were commonly nonsusceptible to cotrimoxazole (33%), followed by ampicillin (20%), while fewer isolates were nonsusceptible to macrolides, represented by azithromycin (10%) [22]. Isolates resistant to chloramphenicol and tetracycline were reported to be less than 10% (9.5%), which is similar to our findings (6% and 7%, respectively) [22]. This is in contrast to a study from Taiwan, where the susceptibility to ampicillin was the lowest among the tested antimicrobials (19.7%), followed by cotrimoxazole (31.1%) [6].

In this study, among ampicillin nonsusceptible *H*. *influenzae* isolates, BLNAR strains were the most frequent compared to BLNAI and BLNASR. Ampicillin nonsusceptible strains have also been reported in various regions among ampicillin nonsusceptible isolates worldwide. In 2016, a study from Japan reported that BLNAR was the most prevalent (60%) among *H*. *influenzae* isolates, followed by BLNAS (17.5%) and BLPAR (10%) [13]. In Korea, one study reported that BLPAR was the most prevalent (47.2%), followed by BLNAS (41.5%) and BLNAR (6.1%) [23]. Meanwhile, a study in Spain reported that BLNAR was the most dominant (56%), followed by BLNAS (17.8%), BLPAR (15.8%), and BLPACR (10.4%) [24]. In this study, we found one isolate that showed resistance to amoxicillin/clavulanate (2 : 1) with an MIC value > 16/8 *μ*g/mL, but was susceptible to ampicillin, defined as BLNACR. Three isolates were resistant to ampicillin/sulbactam, which were defined as BLNASR. In this study, *β*-lactamase-producing strains were defined according to their MIC values and were not confirmed by PCR detection of the gene encoding *β*-lactamase. In conclusion, the prevalence of *H*. *influenzae* carriage in the nasopharynx of school children with AOM in Indonesia was 69.7%. The isolates were predominantly NTHi. The *H*. *influenzae* isolates identified in this study were less susceptible to cotrimoxazole. In addition, BLNAR strains were the most prevalent among the nonsusceptible ampicillin strains.

## Figures and Tables

**Table 1:** Identification and serotyping of 85 *H*. *influenzae* strains isolated from nasopharynx of school children with AOM.

Identification	Children, n (%)
hpd detection	85 (100)
Serotype:	
a	0
b	3 (3.5)
c	0
d	0
e	0
f	1 (1.2)
NTHi	81 (95.3)

**Table 2:** Antimicrobial susceptibility of *H*. *influenzae* isolated from nasopharynx of school children with AOM.

Antimicrobials	Susceptible (%)	Intermediate (%)	Resistant (%)	MIC50 (*µ*g/mL)	MIC90 (*µ*g/mL)	MIC range (*µ*g/mL)
LEVO	100	0	0	≤0.03	≤0.03	≤0.03 to 0.5
CLA	82	18	0	8	16	≤0.12 to 16
FAC	96	1	2	≤4	8	≤4 to >16
AXO	100	0	0	≤0.03	≤0.03	≤0.03 to 0,25
AMP^a^	86	4	11	0,25	4	≤0.12 to >4
FEP	99	0	1	≤0.12	0,25	≤0.12 to >2
SPX	99	0	1	≤0.03	≤0.03	≤0.03 to 0,5
IMI	100	0	0	≤0.5	≤0.5	≤0.5 to 1
SXT	74	7	19	0.25/4.75	>2/38	≤0.06/1.19 to >2/38
MERO	100	0	0	≤0.06	≤0.06	≤0.06 to 0,25
FUR	100	0	0	1	2	≤0.5 to 4
FIX	100	0	0	≤0.12	≤0.12	≤0.12 to 1
TET	93	1	6	0,5	1	≤0.25 to >4
CHL	94	0	6	≤0.5	1	≤0.5 to >4
A/S2	96	0	4	≤1/0.5	≤1/0.5	≤1/0.5 to >2/1
AMC	99	0	1	≤2/1	≤2/1	≤2/1 to >16/8

^a^Breakpoints used for ampicillin: susceptible = ≤1, intermediate = 2, resistant = ≥4. LEVO, levofloxacin; CLA, clarithromycin; FAC, cefaclor; AXO, ceftriaxone; AMP, ampicillin; FEP, cefepime; SPX, sparfloxacin; IMI, imipenem; SXT, trimethoprim/sulfamethoxazole; MERO, meropenem; FUR, cefuroxime; FIX, cefixime; TET, tetracycline; CHL, chloramphenicol; A/S2, ampicillin/sulbactam (2 : 1); AMC, amoxicillin/clavulanic acid (2 : 1).

**Table 3:** Antibiogram of ampicillin nonsusceptible isolates of *H*. *influenzae* isolated from nasopharynx of school children with AOM.

Lab ID	Serotypes	CLA	AMP	A/S2	AMC	AXO	FEP	FUR	FIX	FAC	Grouping
OMP090	NT	S	R	S	S	S	S	S	S	S	BLNAR
OMP094	NT	S	R	S	S	S	S	S	S	S	BLNAR
OMP099	NT	S	R	R	S	S	S	S	S	S	BLNASR
OMP110	NT	S	R	S	S	S	S	S	S	R	BLNAR
OMP124	NT	S	I	S	S	S	S	S	S	I	BLNAI
OMP141	Hi f	S	I	S	S	S	S	S	S	S	BLNAI
OMP151	NT	I	R	S	S	S	S	S	S	S	BLNAR
OMP0158	NT	S	R	R	S	S	S	S	S	S	BLNASR
OMP0161	NT	S	R	S	S	S	S	S	S	S	BLNAR
OMP0179	NT	S	R	S	S	S	S	S	S	S	BLNAR
OMP0200	NT	S	R	R	S	S	S	S	S	R	BLNASR
OMP0119	NT	S	I	S	S	S	S	S	S	S	BLNAI

FAC, cefaclor; CLA, clarithromycin; AXO, ceftriaxone; AMP, ampicillin; FEP, cefepime; FUR, cefuroxime; FIX, cefixime; A/S2, ampicillin/sulbactam (2:1); AMC, amoxicillin/clavulanic acid (2:1). S, susceptible; I, intermediate; R, resistant. BLNAR: *β*-lactamase-nonproducing ampicillin-resistant, BLNASR: *β*-lactamase-nonproducing ampicillin/sulbactam resistant, BLNAI: *β*-lactamase-nonproducing ampicillin intermediate resistant.

## Data Availability

The serotyping and minimum inhibitory concentration data used to support the findings of this study are included within the article.
